# Gardasil^®^ as adjunctive therapy for respiratory papillomatosis at Red Cross Children’s Hospital, Cape Town

**DOI:** 10.4102/jcmsa.v2i1.33

**Published:** 2024-05-24

**Authors:** Shavina Frank, Jessica K. McGuire, Fiona Kabagenyi, Shazia Peer

**Affiliations:** 1 Department of Otorhinolaryngology, Faculty of Health Sciences, University of Cape Town, Cape Town, South Africa; 2 Department of Ear, Nose and Throat, Faculty of Medicine, Makerere University, Kampala, Uganda

**Keywords:** Gardasil-4, Gardasil-4^®^, juvenile-onset recurrent respiratory papilloma, vaccine, upper airway obstruction, tracheostomy, Derkay-Coltrera score, human papilloma virus

## Abstract

**Background:**

Juvenile onset recurrent respiratory papillomatosis (JoRRP) is an incurable condition caused by human papilloma virus (HPV) types 6 and 11, often requiring repeated surgeries and in severe cases, tracheostomy. This imposes a significant socioeconomic burden on patients and families. Gardasil^®^, a proven prophylactic HPV vaccine, is emerging as a potential adjuvant therapy. We studied its response on JoRRP patients at our center.

**Methods:**

We conducted a retrospective review at Red Cross War Memorial Children’s Hospital from January 2015 to June 2022 on histologically confirmed JoRRP cases. Age at diagnosis, baseline and post-dosing Derkay-Coltrera (DC) scores (disease severity measure), inter-surgical intervals and tracheostomy, were collected.

**Results:**

Twenty-five of 30 confirmed cases were included. Average age at diagnosis was 60 months (about 5 years old), with HPV Type 6 in 40% and Type 11 in 48% of patients. All patients received at least one Gardasil^®^ dose, 84% received a second dose and 64% a third dose. Total population DC score decreased from an average of 17 (range: 4-34) pre-first dose to 8 (range: 0-16) after three doses, indicating a 50% reduction. Surgical intervals modestly increased. More significant improvements were seen in patients with aggressive forms of the disease.

**Conclusion:**

This is the first study in Southern Africa highlighting Gardasil^®^ as adjuvant therapy. Despite our limited sample size, new cases observed a linear reduction in DC scores and tracheostomy rates.

**Contribution:**

This suggests that Gardasil^®^ as adjuvant therapy has the potential to reduce disease severity and extend surgical intervals.

## Introduction

Juvenile-onset recurrent respiratory papillomatosis (JoRRP) is a benign disease of the upper aerodigestive tract. The incidence of JoRRP in the developing world is 1.34/100 000 cases per annum.^1^ It is caused by the human papilloma virus (HPV) types 6 and 11, accounting for over 90% of cases. The latter is reportedly associated with a more aggressive disease process. There is currently no cure and repeated surgical clearance of the obstructed airway by exophytic papillomas is standard treatment to alleviate recurrent symptoms. Juvenile onset recurrent respiratory papillomatosis has a varied course and those with rapidly recurrent disease require multiple surgical interventions. A tracheostomy is performed in some patients with ongoing severe upper airway obstruction. Juvenile onset recurrent respiratory papillomatosis has a varied course and those with rapid disease recurrence require multiple surgical interventions and a tracheostomy in some with severe upper airway obstruction. This results in a costly burden to the patient’s family and the healthcare services,^2,3^ notwithstanding the associated significant morbidity and decreased disease-free quality of life years.^2^

Three prophylactic HPV vaccines are currently available: Cervarix^®^ (bivalent), Gardasil^®^ (quadrivalent) and Gardasil^®^ 9 (nonavalent). Although the bivalent vaccines are less costly, they do not cover HPV types 6 and 11.^4^ The quadrivalent Gardasil (Gardasil^®^) vaccine targets the HPV oncogenic types 18, 16 and 6 and 11 (common genotypes that cause JoRRP) and is well established as a prophylactic vaccine.^5^ Emerging data show that it is promising as adjuvant therapy in those with established JoRRP by demonstrating a decrease in disease severity and in repeated surgical clearance.^6^

Our study’s aim was to evaluate the efficacy of Gardasil^®^ vaccine as adjuvant therapy for patients with JoRRP who were managed at our Paediatric ENT Surgery Airway Unit, based at Red Cross War Memorial Children’s Hospital with specific reference to clinical outcomes. Standard demographics, including age at diagnosis and gender, were captured. The primary measure of disease severity was the Derkay-Coltrera Severity (DCS) Scoring System, a universally accepted and standardised grading system for the severity of respiratory papillomatosis at the time of surgical ablation. Derkay-Coltrera Severity includes the sum of clinical and anatomical scores that are quantified numerically to determine disease severity.^7^ Clinical score includes voice quality, stridor and degree of respiratory distress. Anatomical score includes the presence and extent of papillomas at specifically defined subsites of the aerodigestive tract.

Other included parameters were inter-surgical time intervals, tracheostomy insertion and decannulation following Gardasil^®^ therapy. Primary measures were compared between types 6 and 11. HIV test results, where available, were included.

## Research methods and design

### Study design and population

A retrospective hospital chart review of all JORRP patients attending the Paediatric ENT Airway Unit at Red Cross Children’s Hospital in Cape Town, South Africa was done. The study period was from January 2015 to June 2022. All patients with histologically confirmed JoRRP, managed by our unit during this timeline, were given Gardasil^®^ vaccination as adjuvant therapy. Consent was obtained by parents before Gardasil^®^ administration. Age at diagnosis, baseline and post-dosing DCS scores, surgical time intervals, presence of tracheostomy, HPV typing and general demographic factors were recorded. The inclusion criteria included patients with at least one dose of the Gardasil^®^ vaccine administered during the study period. Exclusion criteria included patients already on other adjuvant therapies and those who did receive their first dose of Gardasil^®^ during the study period. The three-dosing regimen from the WHO Advisory Committee on Immunisation Practices 2019 guidelines for prophylactic dosing of Gardasil^®^ was used as a guideline for vaccine administration. The second dose was therefore administered around 2 months and the third dose around 6 months following the initial dose.^8^ It is a standard practice for airway intervention surgery in our unit to administer a proton pump inhibitor (1 mg/kg daily) for 2 weeks post-operatively. This is intended to promote healing following instrumentation of the airway.^9^

### Statistical analysis

Three clinical outcomes were examined in the study. The first was disease severity: baseline and post-dosing DCS scores. We further compared HPV types 6 and 11. The second and third outcomes measured included inter-surgical interval per disease year (grouped: < 10 scopes, 11–20 scopes and > 20 scopes – from diagnosis to end of study period) and the need for tracheostomy. The number of children who were successfully decannulated post-treatment was also reviewed. A paired *t*-test and 95% confidence intervals were used to determine statistical significance. Microsoft^®^ Excel Analysis ToolPak was used.

## Results

Thirty histologically confirmed cases of JoRRP were identified. Five cases were excluded: three received additional Avastin as adjuvant therapy and two did not receive their first dose of the Gardasil^®^ vaccine within the study period. A total of 25 children were included in the study. Demographic factors are tabulated (Table 1). The male-to-female ratio was 3:2. The average age at diagnosis was 60 months (range: 13–113) and 62 months for the first surgery (range: 13–113). All patients underwent HIV testing, with only one patient testing HIV-positive, the remaining 24 tested HIV-negative. A 2-month delay from diagnosis to first surgery occurred in 4 patients. This was attributable to the coronavirus disease 2019 (COVID-19) pandemic and delays in elective operating schedules. All 25 participants included in the study received the first dose of the Gardasil^®^ vaccine, 84% (*n* = 21/25) received a second dose, and 64% (*n* = 16/25) received a third dose. Human papilloma virus type 6 accounted for 40% (*n* = 10/25), type 11 for 48% (*n* = 12/25) and HPV type was unknown in 12% (*n* = 3/25) of the study group. A total of 43% (*n* = 7/16) of patients who received all three doses and 38% (*n* = 8/21) who received at least two doses of the Gardasil^®^ vaccine adhered to our vaccination schedule.

**TABLE 1:** Demographic details of the study sample.

Study cohort of 25 patients	Male	Female	Unknown	Negative	Positive	Unknown
Gender	15	10	0	-	-	-
Outcomes measured	6	11	Unknown	-	-	-
HPV typing	10	12	3	-	-	-
HIV status	-	-	-	24	1	0
Average age D (months)	-	-	-	13	Range D	13–113
Average age FS (months)	-	-	-	26	Range FS	13–113

Note: D, at time of diagnosis; FS, at time of first surgery.

HPV, human papilloma virus.

**FIGURE 1:**
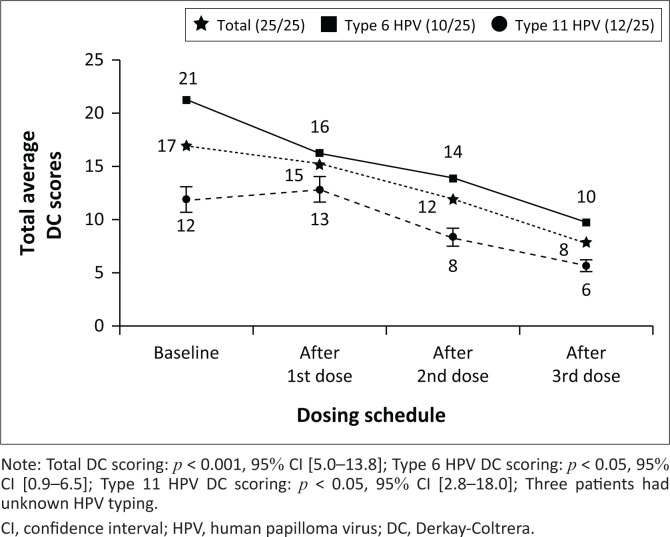
Derkay-Coltrera scores pre- and post-dose of Gardasil^®^ (*n* = 25).

**FIGURE 2:**
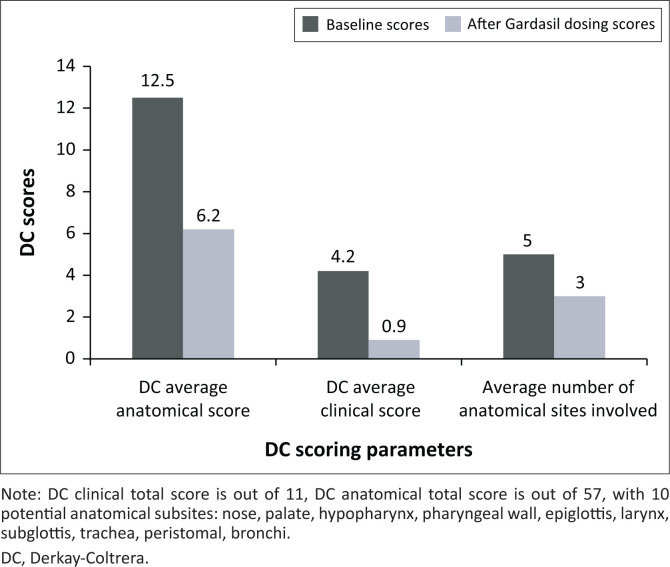
Comparison of disease severity before and after dosing with Gardasil^®^.

There was a statistically significant, linear reduction in the total cohort of Derkay-Coltrera (DC) scores for the sample population (17 pre-dosing to 8 post-dosing completion). A statistically significant decrease in average DC scores was found for HPV type 6, from 12 (pre-dosing) to 6 (post-dosing completion) and type 11, from 21 (pre-dosing) to 10 (post-dosing completion) (Figure 1). Patients presented with an average DCS score of 4.2. DCS clinical scores improved from 4.2 to 0.9 and anatomical scores from 12.5 to 6.2 post dosing of Gardasil^®^ (Figure 2).

Most patients, that is, 19/25 (76%), had < 10 total lifetime scopes during the study period, with 3/25 (12%) having 11–20 total lifetime scopes and 2/25 (8%) having > 20 total lifetime scopes. Both patients in the > 20 total lifetime scopes group were HPV type 11, both were tracheostomy-dependent, and one was decannulated during the study period. Intersurgical intervals marginally increased, with a more notable increase observed in the > 20 total lifetime scopes group (Table 2). A total of 7/25 (28%) was tracheostomy dependent, with 48% found to have HPV type 11 JoRRP. No tracheostomies were performed during the study period, and 4/7 were successfully decannulated during the study period.

**TABLE 2:** Intersurgical interval pre- and post-doses of Gardasil^®^.

Scope category	Baseline	1st Gardasil-4 vaccine	2nd Gardasil-4 vaccine	3rd Gardasil-4 vaccine	*p*
All patients	5.7	6.5	5.5	5.7	0.4130
< 10 scopes	6.9	6.6	5.4	5.0	0.0000*
11–20 scopes	2.4	0.0	7.0	0.0	0.2070
> 20 scopes	2.8	6.0	11.0	9.0	0.0003*

Note: Scope category indicates lifetime scope number up until end of study interval.

* , *p* < 0.001.

## Discussion

Juvenile-onset recurrent respiratory papillomatosis has an unpredictable course, whereby some children with mild disease present with recurrent dysphonia but no obstruction presentation. However, for children with aggressive disease, multiple surgical procedures are the mainstay of treatment to alleviate recurrent obstructive symptoms. In the developing world, the likelihood of requiring a tracheostomy is not trivial. This further increases the health and economic burden per case of JoRRP, which includes repeated surgical procedures, post-surgical follow-ups and non-surgical outpatient surveillance.^10^

This is the first study in Southern Africa to demonstrate an improvement in DC scores after adjuvant treatment with Gardasil^®^, with a more than 50% reduction in DC scores for both types 6 and 11 of JoRRP. No adverse effects were reported by carers of patients who received Gardasil^®^ vaccination.

The average reported age of diagnosis of juvenile-onset RRP is 4 years,^11^ whereas our population was slightly older at 5 years. The JoRRP usually manifests as hoarseness or voice changes in high-income countries.^11^ Yet our patients presented with a DC clinical score of > 4, indicating they presented with airway difficulty in addition to dysphonia. This correlates with South African findings by Seedat et al., who highlighted the difference in clinical presentation in lower and middle income countries (LMICs), where patients present with airway obstruction and preceding hoarseness for a protracted period before seeking medical care.^11^

Meta-analyses by Rosenberg et al. in 2019 and more recently by Goon et al. in 2023, reported an increase in intersurgical intervals following vaccination with Gardasil^®^, thereby highlighting the benefit of Gardasil^®^ as adjuvant therapy in JoRRP.^12,13^ Our study showed a minor overall increase in intersurgical time intervals; the most significant increase was observed in the patients with more severe disease, with an average of 2.8 to 9 months intersurgical intervals following three doses of the Gardasil^®^. In addition, 16/25 patients (64%) received at least one dose of Gardasil^®^ in their 1st year of the disease, with many patients showing an improvement in their DC scores in their first year. Furthermore, none of our patients required a tracheostomy during the study period. Of those already tracheostomy dependent, 75% (4/7) were successfully decannulated during the study period. This has implications for the burden of care as many African countries still report tracheostomy rates of 20% – 51%, and in Nigeria, up to 100%.^11^

Of note, our study has the following limitations: firstly, a small sample size with no control group. Secondly, the study period fell in the COVID-19 pandemic, and the resultant protracted national lockdown period, making it challenging to follow-up patients from out of town, and those who were not in extremis. The scheduled Gardasil^®^ vaccinations may have been affected. Two patients were lost to follow-up during this period. Thirdly, multiple surgeons typically completed the DCS scoring, which could have introduced subjective bias in anatomical scoring. Although not quantifiable, the post-operative use of proton pump inhibitors (PPI) in our patients may have had an influence on outcomes.

## Conclusion

Juvenile-onset recurrent respiratory papillomatosis is a challenging condition to manage for otolaryngologists worldwide as there is no cure. The universally accepted goal is to achieve eradication through prevention measures such as HPV vaccination programmes made available to all children across the world. However, in children already living with the disease, multiple adjuvant therapies have been utilised, including Gardasil^®^. Ours is the first study in Southern Africa to utilise Gardasil^®^ as adjuvant therapy in JoRRP and with a positive clinical outcome. This also shows promise particularly in children who manifest with aggressive disease. Despite a small sample size, a linear reduction in DCS scoring and increased intersurgical intervals (especially in more aggressive disease) were found with the adjuvant use of Gardasil^®^ vaccination. This is evident in both HPV types 6 and 11. However, multicentre collaborative studies with more patients are needed to provide further evidence to support these conclusions.
