# Corrigendum: Gardasil^®^ as adjunctive therapy for respiratory papillomatosis at Red Cross Children’s Hospital, Cape Town

**DOI:** 10.4102/jcmsa.v2i1.93

**Published:** 2024-07-18

**Authors:** Shavina Frank, Jessica K. McGuire, Fiona Kabagenyi, Vincent Pretorius, Shazia Peer

**Affiliations:** 1 Division of Otorhinolaryngology, Department of Surgery, Faculty of Health Sciences, University of Cape Town (Red Cross War Memorial Children’s Hospital), Cape Town, South Africa; 2 Department of Ear Nose and Throat, Faculty of Medicine, Makerere University, Kampala, Uganda

In the published article, Frank S, McGuire JK, Kabagenyi F, Peer S. Gardasil^®^ as adjunctive therapy for respiratory papillomatosis at Red Cross Children’s Hospital, Cape Town. J Coll Med S Afr. 2024;2(1), a33. https://doi.org/10.4102/jcmsa.v2i1.33, Vincent Pretorius was unfortunately left off the list of co-authors in the published article. The corrected authorship list and associated affiliations have been updated. The revised ‘author’s contributions’ statement is as follows:

S.F., F.K., J.K.M., V.P. and S.P. edited and reviewed this article and assisted in developing this research. S.P. supervised this research.

The authors apologise for this error. The correction does not change the study’s findings, its significance or overall interpretation of its results or the scientific conclusions of the article in any way.

